# Effectiveness of intensive stand-alone smoking cessation interventions for individuals with diabetes: A systematic review and intervention component analysis

**DOI:** 10.18332/tid/162329

**Published:** 2023-05-10

**Authors:** Joseph Grech, Ian J. Norman, Roberta Sammut

**Affiliations:** 1 Department of Nursing, Faculty of Health Sciences, University of Malta, Mater Dei Hospital, Msida, Malta; 2 Faculty of Nursing, Midwifery and Palliative Care, King’s College London, London, United Kingdom

**Keywords:** diabetes mellitus, tobacco use disorder, smoking cessation agents, diabetes complications, intervention component analysis

## Abstract

**INTRODUCTION:**

Tobacco smoking poses a significant threat to the health of individuals living with diabetes. Intensive stand-alone smoking cessation interventions, such as multiple or long (>20 minutes) behavioral support sessions focused solely on smoking cessation, with or without the use of pharmacotherapy, increase abstinence when compared to brief advice or usual care in the general population. However, there is limited evidence so far for recommending the use of such interventions amongst individuals with diabetes. This study aimed to assess the effectiveness of intensive stand-alone smoking cessation interventions for individuals living with diabetes and to identify their critical features.

**METHODS:**

A systematic review design with the addition of a pragmatic intervention component analysis using narrative methods was adopted. The key terms ‘diabetes mellitus’ and ‘smoking cessation’ and their synonyms were searched in 15 databases in May 2022. Randomized controlled trials which assessed the effectiveness of intensive stand-alone smoking cessation interventions by comparing them to controls, specifically amongst individuals with diabetes, were included.

**RESULTS:**

A total of 15 articles met the inclusion criteria. Generally, the identified studies reported on the delivery of a multi-component behavioral support smoking cessation intervention for individuals with type I and type II diabetes, providing biochemically verified smoking abstinence rates at follow-up at six months. The overall risk-of-bias of most studies was judged to be of some concern. Despite observing inconsistent findings across the identified studies, interventions consisting of three to four sessions, lasting more than 20 min each, were found to be more likely to be associated with smoking cessation success. The additional use of visual aids depicting diabetes-related complications may also be useful.

**CONCLUSIONS:**

This review provides evidence-based smoking cessation recommendations for use by individuals with diabetes. Nonetheless, given that the findings of some studies were found to be possibly at risk-of-bias, further research to establish the validity of the provided recommendations is suggested.

## INTRODUCTION

Diabetes mellitus is a major public health concern. Diabetes mellitus, which is characterized by chronic high levels of blood glucose, can lead to the development of various macro- and micro-vascular complications, increasing the risk of morbidity and even death^1^. It is estimated that diabetes affects 537 million adults aged 20–79 years worldwide^1^. Tobacco smoking is another major public health problem. While it is well known that tobacco smoking is associated with increased morbidity and mortality in the general population^2^, leading to 8.7 million deaths each year^3^, increasing evidence demonstrates the increased risk of complications and mortality for those who have diabetes and smoke. When compared to non-smokers living with diabetes, both individuals with type I and type II diabetes have been found to have an increased risk for coronary heart disease, myocardial infarction, and stroke, of 54%, 52% and 44%, respectively^4^. A higher risk for cardiovascular mortality and for total mortality for individuals with diabetes who smoke, has also been identified^4^. Tobacco use may also increase the risk of microvascular diabetes complications. While there is insufficient evidence to demonstrate the influence of tobacco use on the development of retinopathy and neuropathy^5^, evidence has shown that smoking increases the risk of diabetic nephropathy amongst both individuals with type I and type II diabetes^6^. Both individuals with type I and type II diabetes who smoke also seem to have poorer cardiometabolic profiles. Non-smokers have been found to have a significant lower HbA1c (a mean difference in HbA1c of -0.61%) and a more favorable lipid profile (an HDL-cholesterol difference of 0.12 mmol/L and an LDL-cholesterol difference of -0.11mmol/L) when compared to smokers.^7^

Smoking cessation, being associated with a significant risk reduction for coronary heart disease^4,8^, and for mortality from cardiovascular disease and total mortality^4^, and better cardiometabolic profiles amongst individuals with diabetes^7^, has been recommended as an essential component of diabetes management. Smoking cessation support, however, can range from a one-off episode of brief tobacco cessation advice or counselling session from a healthcare professional lasting ≤20 min, to more intensive approaches involving multiple and/or longer counselling sessions, with or without additional components, such as the use of pharmacotherapy for smoking cessation (e.g. nicotine replacement therapy NRT, varenicline or buproprion)^9^. Intensive smoking cessation interventions, such as, behavioral support (e.g. counselling) lasting more than >20 min^10^, or interventions that combine behavioral support and pharmacotherapy^11^, or interventions with two or more interacting components, such as multiple long (>20 min) counselling sessions with the addition of pharmacotherapy^12^, have been found to increase smoking cessation success when compared to brief advice or usual care in the general population. Nonetheless, there is limited evidence so far for recommending the use of such intensive interventions amongst individuals with diabetes.

The systematic review and meta-analysis by Nagrebetsky et al.^13^, which compared the effectiveness of intensive stand-alone smoking cessation interventions (i.e. pharmacological and/or non-pharmacological intensive behavioral interventions for smoking cessation which were not part of broader interventions for improving diabetes management) to less intensive interventions (such as usual care or brief smoking cessation advice) for individuals living with diabetes, found no evidence calling for the use of intensive smoking cessation interventions. When compared to less intensive interventions, intensive smoking cessation only resulted in a non-significant increase in biochemically verified smoking abstinence at follow-up at 6 months (RR=1.32; 95% CI: 0.23–7.43)^13^. On the other hand, Zhan et al.^14^ who assessed the effectiveness of psychological interventions for smoking cessation (including both behavioral-based stand-alone smoking cessation interventions or interventions in which smoking cessation was part of a broader intervention for improving diabetes management in their review) comparing these to usual care, found that psychological interventions were more effective in achieving abstinence (RR=2.52; 95% CI: 1.32–4.80). However, the positive observed effect, which was based solely on self-reported data, lasted only up to the follow-up at 3 months^14^. Notwithstanding these inconsistencies, it is worth noting that both reviews suffered from substantial heterogeneity: I^2^=76%^13^ and I^2^=69%^14^, warranting caution in the interpretation of findings and application to clinical practice.

In view of uncertainty about the efficacy of smoking cessation interventions for individuals living with diabetes, a scoping review was recently undertaken to identify the most promising smoking cessation methods amongst such individuals, factoring in the diabetes challenges and barriers to quitting^15^. Grech et al.^15^ mapped the literature on the smoking cessation interventions carried out amongst individuals living with diabetes (including both stand-alone smoking cessation interventions and interventions in which smoking cessation was part of a broader intervention for improving diabetes management) and on the challenges and barriers to smoking cessation that were identified amongst such individuals. Stand-alone smoking cessation interventions were identified as more successful in achieving tobacco abstinence than interventions which included smoking cessation as part of a broader intervention for improving diabetes management^15^. However, given the nature of the review, no specific recommendations as regards the behavioral support methods to use, their intensity (the number of sessions and their duration), and on the use of additional components (apart from suggesting the use of pharmacotherapy for smoking cessation), could be provided^15^, limiting application to clinical practice.

Given the potential of stand-alone smoking cessation interventions in achieving tobacco abstinence amongst individuals with diabetes, an update to the systematic review by Nagrebetsky et al.^13^ was conducted. This review aimed to assess the effectiveness of intensive stand-alone smoking cessation interventions amongst individuals living with diabetes mellitus, and to identify the critical features of the successful interventions.

## METHODS

### Study design

A systematic review of effectiveness, being regarded as the gold standard for identifying evidence-based practice^16^, was best suited for this review. Given that the review also aimed to identify the critical features of the successful interventions, it also included an intervention component-level analysis (ICA) as outlined by Sutcliffe et al.^17^. The ICA by Sutcliffe et al.^17^ is a pragmatic but formal and rigorous approach for analyzing the characteristics of the studied interventions, which may be associated with successful outcomes. This method has been particularly recommended when the studies’ interventions differ from one another, which limits the ability to explore meaningful numbers of mediators and moderators of intervention effect through other formal methods of analysis and synthesis^17^. In view of the significant diversity in the interventions utilized by the study authors (as remarked below), the ICA by Sutcliffe et al.^17^ was found to best suited for this review to provide more information about the critical features of the successful interventions for providing practice recommendations. This review is presented in compliance with the Preferred Reporting Items for Systematic Reviews and Meta-Analysis PRISMA statement (Supplementary file Table 1).

### Inclusion and exclusion criteria

The studies included in this review had to assess the effectiveness of intensive stand-alone smoking cessation interventions by comparing them to a less intensive intervention, such as brief tobacco cessation advice or usual care, specifically amongst individuals with diabetes. Intensive stand-alone interventions included pharmacological and/or non-pharmacological behavioral interventions for smoking cessation, which were not part of broader interventions for improving diabetes management, consisting of multiple and/or long (>20 min) smoking cessation support sessions). Studies in which the experimental smoking cessation intervention was part of a more extensive intervention for diabetes management, such as a lifestyle management intervention for improving diabetes, were thus considered ineligible. The studies included in this review had to include individuals living with (diagnosed) diabetes mellitus as their study population. Studies in which the participants had pre-diabetes or gestational diabetes were deemed ineligible. Furthermore, studies in which only a proportion of the participants had diabetes or reports of studies which were not specific to individuals with diabetes were also excluded. Only published articles, or unpublished reports, of randomized controlled trials were considered in this review. Non-randomized trials were deemed ineligible as these tend to produce higher effect estimates of the studied intervention when compared to randomized trials^18^. Systematic reviews were also not included. This is because none of the identified reviews^13-15^ provided sufficient detail on the critical features of the successful interventions. No language or time limiters were set. The minimum requirement for non-English trials was that the title and/or abstract had to be available in English within the identified (below) bibliographic databases.

### Search strategy

The search was carried out on the 28 May 2022 from inception using the following electronic literature databases: APA PsycInfo, CINAHL Complete, Cochrane Central Register of Controlled Trials, Cochrane Clinical Answers, Cochrane Database of Systematic Reviews, Cochrane Methodology Register, MEDLINE Complete, ProQuest Dissertations & Theses A&I, Public Health Database, PubMed, Scopus, System for Information on Grey Literature in Europe, and all the databases on Web of Science. Based on the review objective, the main keywords, ‘diabetes mellitus’ and ‘smoking cessation’ and their synonyms (Supplementary file Table 2), were combined using the Boolean operators ‘AND’ and ‘OR’, and searched in titles, abstracts, and subject headings/medical subject headings accordingly. The search strategy used for searching in Web of Science is outlined in Supplementary file Table 3.

### Study selection

After carrying out the search, the identified records were collated on Mendeley® for de-duplication. The remaining records were screened by reading titles and abstracts. Potentially relevant articles were then read and assessed for eligibility basing decisions on the inclusion and exclusion criteria. The reference lists of these studies and those of the identified reviews^13-15^ were also examined for the identification of other possible suitable studies for inclusion in this systematic review.

### Data extraction

The following information was extracted from all the identified studies: authors; year of study; location; study duration; detailed information on the experimental smoking cessation intervention, its components and the control intervention; study sample; percentage followed up; smoking cessation outcome, time-points and reporting methods; information about the researchers’ reflections and accounts of their experience in evaluating the intervention for conducting the ICA as per Sutcliffe et al.^17^; and other relevant observations. Furthermore, the information required to assess risk-of-bias (as outlined in the Cochrane risk-of-bias tool, RoB 2)^19^ for each of the identified studies, was also extracted.

### Quality assessment

In this review, the RoB 2 for randomized parallel-group trials and the RoB 2 tool specific to cluster-randomized trials were utilized. Studies found to be at high risk-of-bias were not excluded from the review or from the analysis. As recommended by Higgins et al.^20^, the information obtained in carrying out the risk-of-bias assessment was presented as part of the review’s findings and was also considered in the analysis and conclusions of the review. The *robvis* tool^21^ was used to visualize the risk-of-bias assessments.

### Synthesis of results

A meta-analysis of effect estimates was initially the preferred method of synthesis. However, given the significant diversity in the interventions (both experimental and control interventions) utilized by the study authors, and the incomplete reporting of outcomes/effect estimates in some of the identified studies^22-25^, this was not recommended^18,26^. Thus, following data extraction and charting of data in table format, a narrative approach using vote counting based on the direction of effect^18^, was adopted to analyze the results. In essence, the studies showing a statistically significant increase in smoking abstinence were compared to the studies which did not. Additionally, a narrative component analysis, in which the interventions’ critical features were identified by following the method outlined by Sutcliffe et al.^17^, followed. This included the mapping of the characteristics of the interventions, taking into consideration the effectiveness of the interventions for establishing the components which appeared to be of significance, and the coding of informal data on the researchers’ reflections in evaluating the intervention (using inductive thematic analysis), to help understand the association between the identified characteristics and the studies outcomes.

## RESULTS

### Search results

**Figure 1:**
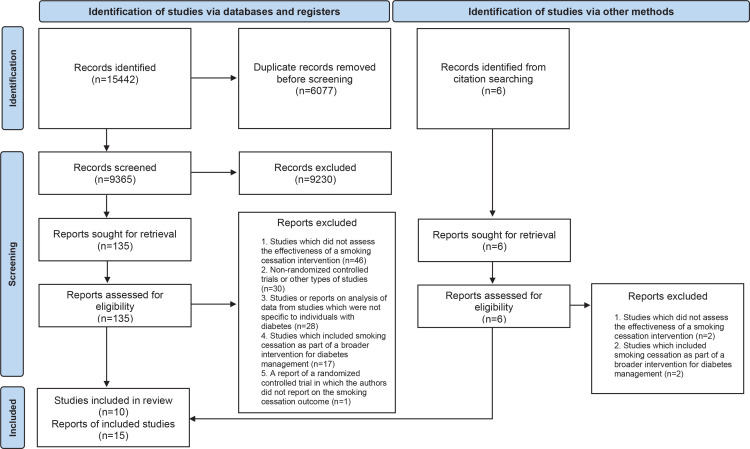
PRISMA flow diagram

The PRISMA 2020 flow diagram^27^ was utilized to outline the selection process, providing details on the exclusion reasons at the full-text level of screening (Figure 1). A total of 15442 records were retrieved of which 6007 were found to be duplicates. After removing duplicates, 9365 were screened by reading titles and abstracts. A total of 135 reports were found to be possibly relevant and were screened at full-text level. On matching these publications to the inclusion criteria, 122 reports were found to be ineligible as they did not report on the effectiveness of stand-alone smoking cessation interventions for individuals with diabetes from randomized controlled trials. Conversely, 13 reports were deemed eligible. An additional six reports, which were obtained from citation searching, were assessed for eligibility. Two reports were found to be eligible and were included. This led to a final selection of 15 reports.

Some of the identified articles reported the same study, resulting in a smaller number of studies. Both Lam et al.^28^ and Thankappan et al.^29^ published the findings from the randomized control trials by Li et al.^30^ and Thankappan et al.^31^, respectively, in conference proceedings. Furthermore, in the publications by Thankappan et al.^25^ and Nichter et al.^24^, the authors followed up participants from the Thankappan et al.^31^ trial for a total of one and two years, respectively, without providing any additional interventions. Additionally, Mini et al.^23^ reported on the biochemical verification of a sample of non-smokers who participated in the Thankappan et al.^31^ trail at follow-up at 1 year, in their publication. Thus, the total number of studies included was 10.

### Characteristics of the selected studies and relevant findings

The characteristics and the relevant findings of the identified studies are outlined in Table 1. Except for the study by Pérez-Tortosa et al.^32^, who reported the findings from a cluster randomized parallel-group trial, all the remaining publications reported the findings from individually randomized parallel-group trials. All studies were published in journals except for the study by Albaroodi et al.^33^ which was available as a preprint.

**Table 1:** Characteristics of the identified trials and the reported smoking cessation outcome at six months and other relevant findings

*Authors Date*	*Country*	*Sample characteristics^a^*	*Experimental intervention*	*Control intervention*	*Study setting Provider*	*Follow-up period (months)*	*Percentage followed up*	*Biochemically verified smoking abstinence at follow-up at 6 months*		*Other relevant findings/comments*
								*Intervention n (%)*	*Control n (%)*	
Albaroodi et al.^33^ 2021	Malaysia	n=140 T1DM=13 T2DM=35 unknown=78 mean age: 47.6±13.6 years 95.2% male participants	Three (5-min) counselling sessions based on the 5As algorithm over three to four months	Routine care	Diabetes clinic Physicians and nurses	6	90	4	4	Preprint – not peer reviewed.
Ardron et al.^34^ 1988	England	n=60 T1DM=50 T2DM=10 mean age: 29.1±7.4 years 48.3% male participants	Brief advice (5-min) Counselling (lengthier session) Smoking cessation leaflet Home visit within two weeks	Brief advice	Diabetes clinic Medical registrar and diabetes home visitor	6	100	0	1	
Canga et al.^35^ 2000	Spain	n=280 T1DM=85 T2DM=195 mean age: 55±15.0 years 86.0% male participants	Counselling session (40-min) Self-help written material Five follow-up contacts (a letter, a phone call or a visit) NRT accordingly	Usual care	Primary care centers and hospitals Nurse	6	99.3	25 (17.0)	3 (2.3)	
Fowler et al.^22^ 1989	England	n=34 T1DM=12 T2DM=22 mean age of newly diagnosed (ND) patients: 47±9.0 years Those with pre-existing (PE) diabetes: 53±13.0 years	Intervention for ND patients and those with PE diabetes: Four (30-min) educational visits over six months Use of visual aids of diabetic related complications	Intervention for ND patients: usual care with late access to the intervention. Intervention for patients with PE diabetes: counselling sessions	Diabetes clinic Health professionals	6	100			It is not known in which group the smokers who quit smoking (n=3) pertained. Drop-outs from the program were high.
Hokanson et al.^37^ 2006	United States	n=114 (T2DM) mean age: 54±9.0 years 57.0% male participants	Counselling based on motivational interviewing (MI) – initial 20 minutes session and three to six 10-min telephone sessions NRT or bupropion accordingly	Information about cessation programs	Diabetes center Nurse	6	63.2	6 (16)	6 (17)	
Lam et al.^28^ 2017 Li et al.^30^ 2017	China	n=557 (T2DM) mean age: 56±11.4 years 88.3% male participants	A 20-min counselling session based on the 5As algorithm and tailored to the participants’ stage of change Booster sessions at one week and one month Self-help smoking cessation leaflet Diabetes specific smoking cessation leaflet	Usual care Brief smoking cessation advice Self-help smoking cessation leaflet	Diabetic clinics Nurse counsellor	12	79.1	38 (13.4)^b^	39 (14.2)^b^	At follow-up at 12 months, 9 (3.2%) vs 14 (5.1%) participants from the intervention and control group, respectively, were abstinent from smoking (biochemically verified; p=0.25).
Ng et al.^38^ 2010	Indonesia	n=71 (T2DM) mean age: 56±9.0 years 100% male participants	Counselling session (30-min) based on the 5As algorithm Control intervention	Brief advice using visual aids of smoking associated diabetic complications Educational materials on the smoking associated diabetic harm	Diabetic clinics and smoking cessation clinic Doctor and counsellor	6	78.9	14 (36.8)^b^	10 (30.3)^b^	A significant decrease in smoking prevalence in both groups.
Pérez-Tortosa et al.^32^ 2015	Spain	n=948 (T1DM and T2DM) mean age: 59.7±11.3 years 75.7% male participants	Counselling sessions based on MI and participants’ stage of change (median – four 22.1-min visits) Pharmacotherapy	Usual care	Primary care practice GPs and nurses	12	76.2			At follow-up at12 months, 67 (17.8%) vs 90 (26.1%) participants from the control and intervention group, respectively, were found to be abstinent from smoking (biochemically verified; p=0.007).
Sawicki et al.^36^ 1993	Ireland	n=89 T1DM=72 T2DM=17 mean age: 38±12.0 years 61% male participants	Ten weekly (90-min) behavioral support sessions NRT accordingly	Brief advice NRT accordingly	Diabetes clinic Psychotherapist	6	100	2	7	Only 25 (57%) attended the support sessions.
Thankappan et al.^25,29,31^ 2013, 2014 Mini et al.^23^ 2015 Nichter et al.^24^ 2018	India	n=224 mean age: 53 years 100% male participants	Three counselling sessions (30-min each) based on the 5As algorithm at baseline, at one month and at three months Control intervention	Brief advice using visual aids of smoking-associated diabetic complications Educational materials on the smoking-associated diabetic harm	Diabetic clinics Doctor and counsellor	6–24	87.5	58 (51.8)^b^	14 (12.5)^b^	Adjusted odds ratio at 6 months: (AOR=8.4; 95% CI: 4.1–17.1) at 12 months: (AOR=3.35; 95% CI:1.82–6.18) (self-reported data were confirmed in 86%) at 24 months: only five were abstinent.

a T1DM: type I diabetes. T2DM: type II diabetes.

b Self-reported data. MI: motivational interviewing. GPs: general practitioners.

Most studies included individuals with both type I and type II diabetes as study participants^33-36^, who were mostly men^30-33,35-38^, and in their fifties^30-32,35,37,38^. Sample sizes varied across the studies; from n=34^22^ to n=948^32^; however, only three studies^30,32,35^ reported *a priori* power calculations to detect a significance in smoking cessation outcome.

Most authors based their intervention on the 5As algorithm (Ask, Advise, Assess, Assist and Arrange)^30,31,33,38^, followed by motivational interviewing^32,37^; however, the frequency and the number of sessions provided, and their duration greatly varied across the studies. In two studies^30,32^, the authors also took into consideration the participants’ current stage of behavior as per the trans-theoretical model of change^39^ when delivering the intervention. Additionally in four of the identified studies^32,35-37^, NRT or buproprion were provided depending on the set eligibility criteria. Information material, such as general or diabetes-specific smoking cessation leaflets were also provided in four studies^30,31,34,38^, while in the studies of Fowler et al.^22^, Ng et al.^38^ and Thankappan et al.^31^, visual aids of diabetes-related complications were also utilized. The identified interventions were mostly delivered by nurses^30,32,33,35,37^, and doctors^31-34,38^, and delivered in diabetes centers/clinics^22,30,31,33,36-38^.

As seen in Table 2, in most studies the authors assessed the impact of the studied intervention on smoking cessation for up to 6 months. Smoking abstinence was usually defined as a 7-day point prevalence abstinence^25,30,31,37,38^, and biochemically verified (at the end of the study) by measuring exhaled carbon monoxide^30,32-34^, and/or cotinine in saliva, urine or blood plasma^22,30,34-37^.

**Table 2:** Main components of the smoking cessation interventions of the included studies

*Study (studies with non-significant findings in italics)*	*Length of session*		*Number of sessions provided*			*Additional information on tobacco-associated diabetic complications*		*Provision of pharmaco-therapy*	*General stop-smoking informational material*
	*≤20 min*	*>20 min*	*1–2*	*3–4*	*≥5*	*Visual aids*	*Leaflets*		
**Intervention based on the 5As framework**									
Albaroodi et al.^33^ (2021)	√			√					
Li et al.^30^ (2017)	√			√			√		√
Ng et al.^38^ (2010)^a,b^		√	√			√	√		
Thankappan et al.^31^ (2013)^a^		√		√		√	√		
**Intervention based on motivational interviewing**									
Hokanson et al.^37^ (2006)	√			√				√	
Pérez-Tortosa et al.^32^ (2015)		√		√				√	
**Intervention not based on a specific framework**									
Ardron et al.^34^ (1988)^a^		√	√						√
Canga et al.^35^ (2000)		√		√				√	√
Fowler et al.^22^ (1989)		√		√		√			
Sawicki et al.^36^ (1993)		√			√			√	

a Included brief smoking cessation advice prior to the intensive session(s).

b Observed a significant decrease in the self-reported smoking prevalence in both groups.

### Assessment for risk-of-bias

**Figure 2:**
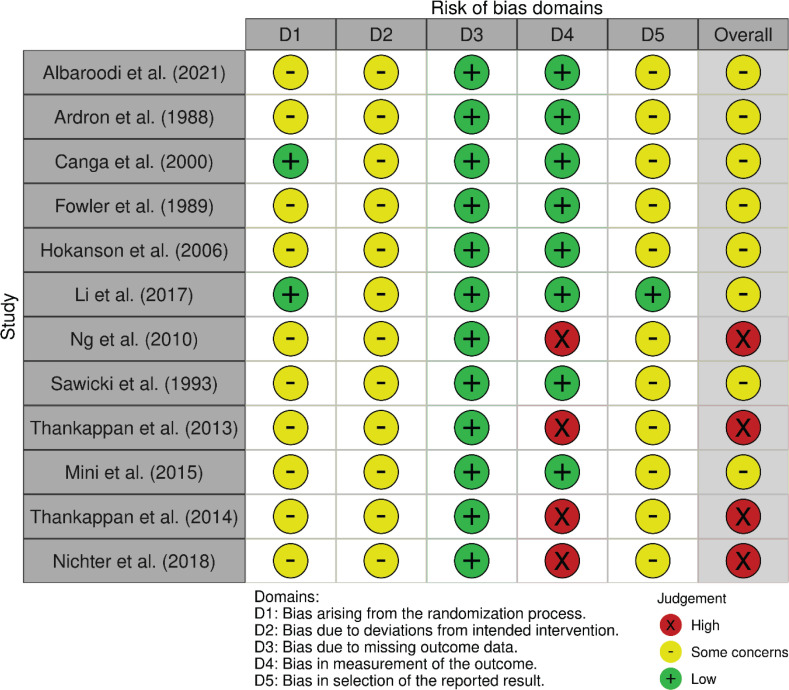
Summary of risk-of-bias assessments performed using the RoB 2 for randomized parallel-group trials

**Figure 3:**
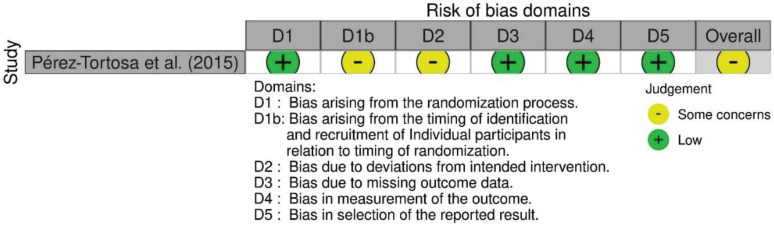
Summary of risk-of-bias assessment performed using the RoB 2 for cluster-randomized trials

A risk-of-bias assessment was carried out on the endpoint reported smoking cessation outcome of each publication. Risk-of-bias assessments were carried out by following the guide by Higgins et al.^20^. The assessments using RoB 2 are shown in Figure 2, while the risk-of-bias assessment using the RoB 2 tool for cluster-randomized trials is shown in Figure 3. Given that all studies were judged to be of concern of risk-of-bias in at least one domain, the overall risk-of-bias of most studies was also judged to be of some concern. Conversely, the overall risk-of-bias of the reports by Ng et al.^38^, Thankappan et al.^25,31^ and Nichter et al.^24^ was judged ‘high’, as these were found to be at high risk-of-bias in the measurement of the smoking cessation outcome, being based on self-reported data.

### Narrative analysis of the findings of the studies

As outlined in Table 1, significant differences in smoking cessation between the intervention and control group were only reported in the studies by Canga et al.^35^ and Thankappan et al.^31^ at follow-up at 6 months, and in the studies by Pérez-Tortosa et al.^32^ and Thankappan et al.^25^ at follow-up at 1 year. Conversely, the other study authors^22,24,30,33,34,36-38^ did not report a significant improvement in the smoking cessation rate of the intervention group when compared to the control group; and although Ng et al.^38^ reported a significant decrease in the self-reported smoking prevalence in both groups at follow-up at 6 months, in these studies the smoking abstinence rate in both the intervention and control group was also deemed to be insignificant. Given these inconsistent findings, and taking into consideration the high level of heterogeneity amongst the interventions of the studies, a narrative component analysis was conducted.

### Narrative component analysis

Guided by Sutcliffe et al.^17^, the main features of the interventions were mapped out at the following levels: the framework/behavioral support method on which the intervention was based; its intensity, the length of each session and the number of sessions provided; the provision of additional information about tobacco associated diabetic complications, using visual aids or leaflets; the provision of pharmacotherapy; and the provision of general stop-smoking information material. Given that in most studies^22,31-34,38^ two or more health professionals were involved in delivering the intervention, the interventions of the studies were not mapped at the provider level. However, the health professionals involved in providing the successful features of the interventions were then identified. Table 2 maps out the characteristics of the interventions of each study based on these categories.

It appears that intensive smoking cessation interventions may enhance smoking cessation success. Canga et al.^35^, Thankappan et al.^31^ and Pérez-Tortosa et al.^32^, who provided 3–4 sessions of duration >20 min for their study participants, found that more smokers in the intervention group quit smoking when compared to those in the control group. Conversely, in the other studies, in which a non-significant smoking cessation outcome was reported, the experimental intervention was brief ≤20 min^30,33,37^ or consisted of only one or two lengthier sessions in total^34,38^. While for the studies of Fowler et al.^22^ and Sawicki et al.^36^, whose experimental intervention was of an intensive nature, reported a non-significant outcome, it is worth noting that few participants adhered to the study protocol, possibly undermining the intervention’s effectiveness. Trained nurses^32,35^, doctors^32^ and counsellors/diabetes educators^31^ provided the lengthier sessions as part of the successful smoking cessation interventions.

The provision of frequent smoking cessation support also seems to have been beneficial. In the studies of Canga et al.^35^ and Pérez-Tortosa et al.^32^, smokers who were ready to quit were provided with frequent smoking cessation support (follow-up appointments at 1 to 2 weeks). Given that the frequency of the sessions varied across the identified studies, it is, however, difficult to establish the ideal total duration of the studied interventions in terms of months.

There is, however, not enough evidence to recommend the use of a specific framework/method for smoking cessation. While both Ng et al.^38^ and Thankappan et al.^31^, who based their intervention on the 5As framework, reported a significant decrease in smoking prevalence in their studies, Albaroodi et al.^33^ and Li et al.^30^, who also utilized the same framework, did not. Similarly, while Pérez-Tortosa et al.^32^, whose intervention was based on motivational interviewing (MI), reported significant findings, Hokanson et al.^37^, who utilized the same technique, did not. There is also not enough evidence to recommend the tailoring of interventions based on the participants’ stage of change, as unlike Pérez-Tortosa et al.^32^, Li et al.^30^, who also based their intervention according to the participants’ stage of change, did not report a significant smoking cessation outcome.

It is not clear whether the addition of pharmacotherapy to behavioral support helped increase smoking cessation success. While both Pérez-Tortosa et al.^32^ and Canga et al.^35^, who provided pharmacotherapy for smoking cessation to those assigned to the intervention group observed a significant smoking cessation outcome, they did not report the smoking cessation rate of those who used it. It is also worth noting that in the study of Canga et al.^35^, only 25 out of 105 participants utilized the provided NRT. On the other hand, it is even more difficult to ascertain the effect of the provision of pharmacotherapy on smoking cessation in the Hokanson et al.^37^ and Sawicki et al.^36^ studies. This is because in both studies the participants in the intervention and control groups made use of such treatment.

On the other hand, the use of visual aids of diabetes-related complications, may have been useful in supporting smoking cessation. When considering that in the study of Fowler et al.^22^ few participants adhered to the study protocol, possibly undermining the intervention’s effectiveness, in both Ng et al.^38^ and Thankappan et al.^31^, who also used visual aids of diabetes-related complications, a significant decrease in smoking prevalence was reported. Nonetheless, the use of diabetes-specific leaflets, in which information on diabetes-related complications was also conveyed, is not clear, as unlike Ng et al.^38^ and Thankappan et al.^31^, Li et al.^30^ did not report a significant smoking cessation outcome.

There is not enough evidence to suggest the use of general stop-smoking leaflets. Unlike the Canga et al.^35^ study, in both Ardron et al.^34^ and Li et al.^30^ (the latter of which provided leaflets to both the intervention and control group), non-significant findings were reported.

### Analysis of informal evidence

As part of the ICA outlined by Sutcliffe et al.^17^, the researchers’ reflections, and accounts of their experience in evaluating the intervention, were coded to help understand the association between the identified intervention features and the success or failure of the interventions. Two major themes were identified: ‘intensive smoking cessation support’, and ‘strong warning messages on tobacco associated diabetic complications’, both of which were identified as being associated with smoking cessation success. Table 3 outlines these themes, providing examples of the underlying evidence, the number of studies contributing to these themes, and a brief explanation of the association between these themes and the studies’ outcomes.

**Table 3:** Identified themes from the researchers’ reflections and accounts of their experience

*Theme*	*Number of studies contributing evidence to the theme*	*Informal evidence example*	*Correspondence between theme and study outcomes*
**Intensive smoking cessation support**	6	‘It is thus of paramount importance to design intensive … interventions.’ (Li et al.^30^)	Canga et al.^35^, Pérez-Tortosa et al.^32^ and Thankappan et al.^31^ acknowledged the significance of an intensive smoking cessation intervention in achieving
		‘An intensive intervention adapted to the individual stage of change delivered in primary care for diabetic smokers was feasible and effective.’ (Pérez-Tortosa et al.^32^)	the outlined results. On the other hand, Albaroodi et al.^33^, Li et al.^30^ and Hokanson et al.^37^ whose interventions were less intensive in nature and
		‘This study found a dose response relationship between counseling and quit rate.’ (Thankappan et al.^31^)	unsuccessful, remarked on the need for a more intensive intervention.
**Strong warning messages on tobacco associated diabetic complications**	3	‘Our findings suggest that a brief disease-centered cessation message from the doctor, given in conjunction with use of disease-complication visual aids, has a significant impact on diabetes patients.’ (Ng et al.^38^)	Both Ng et al.^38^ and Thankappan et al.^31^ whose interventions included strong warning messages on tobacco-associated diabetic complications (such as visual aids), reported a significant decrease in smoking prevalence.
		‘In our study both the doctor and the counselor used visual aids and diabetes specific smoking cessation materials ... to motivate patients to consider quitting to prevent complications from diabetes.’ (Thankappan et al.^31^)	Conversely, Li et al.^30^ whose findings were not significant, recommended the use of stronger messages on tobacco-associated diabetic complications to promote smoking cessation.

## DISCUSSION

Similar to the systematic review and meta-analysis by Nagrebetsky et al.^13^, this systematic review reports inconsistent findings across the identified studies. The relatively small number of trials identified, some of which were under powered, and the significant diversity in the interventions utilized by the study authors, limiting comparability, limited the ability to establish the effectiveness of stand-alone smoking cessation interventions for use amongst individuals with diabetes. Nonetheless, the addition of an ICA provided more evidence-based recommendations for smoking cessation practice than the previous reviews^13,15^, as it helped to identify some of the ‘active ingredients’ of the identified diverse multi-component smoking cessation interventions.

Intensive smoking cessation interventions may help enhance smoking cessation success. The smoking cessation interventions which consisted of three to four sessions, lasting >20 min each, were generally more successful. Additionally, the provision of frequent smoking cessation support also seems to be beneficial. These findings are in line with current evidence on the effectiveness of smoking cessation interventions amongst the general population^10,12^.

The use of visual aids depicting diabetes-related complications, may also prove to be useful in supporting smoking cessation. Pictorial warnings of tobacco related complications have in fact been found to elicit negative smoking attitudes and increase intentions to stop smoking^40^. Given that the literature reports that some individuals with diabetes tend to be unconvinced about the additional risks posed by tobacco use on their health,^15^ the use of such strong warnings as part of diabetes-specific intensive smoking cessation support efforts is further recommended.

Conversely, there is not enough evidence to suggest the use of diabetes-specific or general stop-smoking informational material. However, this is in line with current evidence, as in their systematic review and meta-analysis Livingstone-Banks et al.^41^ also found that there is no evidence that informational material increases the effectiveness of smoking cessation advice from a health professional or in using NRT in the general population (RR=0.99; 95% CI: 0.76–1.28).

There is also limited evidence to suggest the use of a specific framework or behavior change method for smoking cessation amongst individuals with diabetes. As was highlighted in the systematic review and meta-analysis by Lindson et al.^42^, who evaluated the efficacy of MI smoking cessation interventions amongst the general population, this review also reports inconsistent findings on the use of MI-based smoking cessation interventions amongst individuals with diabetes. It was also observed that in the study by Pérez-Tortosa et al.^32^, who reported a significant smoking cessation outcome on using MI, and its study protocol^43^, the authors provided almost no detail on the structure or components of the MI-based intervention which was used. Given that MI-based smoking cessation interventions have been found to vary at large^42^, the poor reporting in the Pérez-Tortosa et al.^32^ study limits further the ability to draw any conclusions on the use of MI-based smoking cessation interventions amongst individuals with diabetes.

Similar to what was reported in the systematic review and meta-analysis by Cahill et al.^44^, who assessed the efficacy of stage-based smoking cessation intervention amongst the general population, there is also not enough evidence to suggest the use of stage-based smoking cessation interventions amongst individuals with diabetes. The classification of participants based on the stages of change as per the trans-theoretical model of change^39^ for subsequent tailoring of smoking cessation support, has in fact been long questioned and also discouraged^45,46^. Furthermore, as was observed in the study of Pérez-Tortosa et al.^32^, who tailored their intervention to the participants’ stage of change, in taking the precontemplation stage as a reference point showed that being in the contemplation or preparation/action stage at baseline rather decreased the odds of quitting smoking, which should have not been the case. Unlike the smokers in the precontemplation stage, defined as having no intention to quit smoking in the next 6 months, the smokers in the contemplation or preparation/action stage indicated their intention to quit smoking in the next 6 months and next 30 days or were currently quitting, respectively^39^. Given that the provision of a comprehensive intensive smoking cessation intervention for those in the precontemplation stage, which first aimed to motivate and encourage them to quit smoking, and then supported them towards quitting, was more likely to increase smoking cessation success, the use of a rigid tailored approach based on the assumed participants’ stage of change is rather not recommended for use amongst individuals with diabetes.

While the 5As algorithm for smoking cessation has been featured in guidelines for treating tobacco dependence in the general population^47^, and suggested as a framework for the provision of both brief and intensive smoking interventions amongst individuals with diabetes^48^, the underpinning evidence for recommending this practice was still found to be limited. Future research is thus required to recommend the use of the 5As framework for smoking cessation amongst individuals with diabetes.

Despite the promising use of pharmacotherapy for smoking cessation amongst individuals with diabetes^15^, this review could not establish its significance. Given that the effectiveness of the use of pharmacotherapy for smoking cessation amongst the general population has been highlighted in the literature^11,12^, further research is also required amongst this specific population.

### Strengths and limitations

This review builds on the findings of the scoping review by Grech et al.^15^, assessing the effectiveness of intensive stand-alone smoking cessation interventions amongst individuals with diabetes. The strength of this review was that it comprised a systematic search of randomized trials of stand-alone smoking cessation interventions for individuals with diabetes, utilizing a wide range of databases. This review does not include trials in which smoking cessation was a part of a more extensive intervention for diabetes management. Furthermore, studies in which only a proportion of the participants had diabetes or reports of studies which were not specific to individuals with diabetes, were also excluded. While this limited the number of trials to be reviewed, it allowed us to specifically measure the effect of stand-alone smoking cessation interventions which were specifically designed for and delivered to individuals with diabetes.

While a meta-analysis of effect estimates was the initial preferred method of synthesis, given the significant diversity in the interventions utilized by the study authors, and the incomplete reporting of outcomes/effect estimates in some of the identified studies^22-25^, this was not recommended. Nonetheless, the addition of an ICA to the systematic review proved insightful, as in analyzing the components of the identified diverse interventions some critical features of the successful interventions were identified. Furthermore, recommendations for further research were also provided.

Despite the utility of the ICA to this systematic review, it is worth noting that some of the findings of the studies were found to be possibly at risk-of-bias. Thus, further research to establish the validity of these findings is recommended. Sutcliffe et al.^17^ suggest carrying out qualitative research to explore the views and experiences of recipients and providers of the identified features. Apart from establishing the validity of the obtained findings^17^, in carrying out qualitative research with such stakeholders, the need for other smoking cessation intervention characteristics (specific to individuals with diabetes), may be identified. Given that healthcare interventions are very much dependent on patient involvement and their attitudes to them^49^, the exploration of the views of individuals with diabetes on the identified promising smoking cessation components and their perceived needs to quit smoking, is thus recommended.

## CONCLUSIONS

Tobacco smoking poses a significant threat to the health of those living with diabetes. Given the lack of evidence-based smoking cessation recommendations for use amongst individuals with diabetes, this systematic review aimed to assess the effectiveness of intensive stand-alone smoking cessation interventions amongst such individuals, and to identify the critical features of the successful interventions.

Despite observing inconsistent findings across the identified studies, limiting the ability to establish the effectiveness of intensive stand-alone smoking cessation interventions for use amongst individuals with diabetes, the addition of an ICA proved useful as it helped to identify some of the critical features of the successful interventions, providing evidence-based practice recommendations. Intensive smoking cessation interventions were generally more likely to be associated with smoking cessation success. Smoking cessation interventions which consisted of three to four sessions, lasting >20 min each, were generally more successful. The provision of frequent smoking cessation support was also found to be of possible significance. Additionally, the use of visual aids depicting diabetes-related complications may also have helped in supporting smoking cessation efforts. On the other hand, further research is required to recommend the use of the 5As framework for smoking cessation and to establish the significance of the use of pharmacotherapy for smoking cessation amongst individuals with diabetes. To establish the validity of this review’s findings, the exploration of the views of individuals with diabetes on the identified promising smoking cessation components is also recommended.

## Data Availability

The data supporting this research are available from the authors on reasonable request.
